# Electrohydrodynamic printing technology: mechanisms, control, and applications

**DOI:** 10.1038/s41378-026-01195-3

**Published:** 2026-03-10

**Authors:** Yidong Tian, Jiajun Zhou, Hengjia Zhu, Kaiwen Huo, Xianli Xie, Wei Zhang, Huai Zheng

**Affiliations:** 1 School of Power and Mechanical Engineering, Wuhan University, Wuhan, China; 2 State Key Laboratory of Intelligent Vehicle Safety Technology, Chongqing Changan Automobile Company Limited, Chongqing, China; 3 Aeronautical Engineering Institute, Civil Aviation University of China, Tianjin, China

**Keywords:** Nanobiotechnology, Nanoscale materials, Nanoscale devices

## Abstract

Electrohydrodynamic (EHD) printing is an advanced micro/nanoscale additive manufacturing technique. Owing to its high-resolution capability, broad material compatibility, diverse printing modes, and low cost, it has attracted widespread attention. Nevertheless, significant challenges remain in transitioning EHD printing from the laboratory to large-scale industrial production. This paper elucidates the mechanisms of EHD printing and details control methods for high-resolution, controllable micro/nanopattern fabrication, including process-parameter optimization, rheological design of functional inks, and innovations in system architecture. We summarize recent applications in electronic devices, biomedicine, and optical components, and discuss development directions and prospects for industrial adoption.

## Introduction

With the rapid development of advanced manufacturing, micro/nanoscale processing has become a research hotspot^1–3^. Subtractive methods, like photolithography and plasma beam etching, face limitations in creating intricate 3D structures due to their layer-by-layer material removal approach^4–6^. EHD printing provides a promising alternative. By applying strong electric fields, this method directly draws micro/nano jets from polarizable solutions to manufacture complex structures. The process operates in confined spaces, requires no contact, and eliminates the need for masks or molds, providing a novel solution for micro/nano fabrication^7^.

The investigation of fluid flow under electric fields can be traced back to the 17th century, but it was not until 1917 that Zeleny first captured images of fluid jetting and breakup under electric fields, marking the formal beginning of EHD research^8,9^. The high-voltage electrospinning process was first proposed in 1934, though it received little attention at the time. In 1969, Taylor^10^ introduced the concept of the “Taylor cone” to describe the unique shape of charged liquid menisci and proposed a method for calculating the critical cone angle. Around 1995, the application of electrospray technology attracted more scholars to investigate the mechanisms of EHD printing^11^. In 2007, Rogers’ team^12^ applied EHD printing to electronic device manufacturing, successfully fabricating ring oscillators and TFTs using various types of inks, sparking a surge of research in this field. The details of the abbreviations are shown in Table 1. The mutual promotion between applications and fundamental research has since driven the rapid development, with nearly a thousand related papers now published annually.

**Table 1:** List of abbreviations

Abbreviation	Full-title
DOD	Drop-on-demand
EHD	Electrohydrodynamic
ILC	Iterative learning control
ML	Machine learning
MLA	Microlens array
MSC	Micro-supercapacitor
TENG	Triboelectric nanogenerator
TFT	Thin-film transistor
RP	Redox printing
VOF	Volume of fluid

EHD printing leverages electric fields to form Taylor cones, capable of both continuous jetting to fabricate fiber structures and jet fragmentation to generate micro-droplets. Excellent ink versatility and nanoscale ultra-high resolution are also key advantages^13–15^. The permissible viscosity of functional inks significantly exceeds that of inkjet printing, accommodating a diverse range of materials, including metals, carbon-based materials, organic semiconductors, and various polymers. It provides breakthrough solutions for fields such as flexible electronics^16,17^, biomedical applications^18,19^, and microsensors^20,21^. This technology has currently achieved early industrial applications and realized nanoscale precision printing at laboratory^22–24^.

Despite the immense potential of EHD printing in micro/nanofabrication, achieving high-resolution, high-consistency industrial applications still faces severe performance control challenges. Due to the nonlinear coupling of multi-physics fields, including fluid mechanics, charge dynamics, and solvent thermodynamics, the system exhibits high sensitivity to environmental fluctuations and process parameter variations. Multi-dimensional control strategies have been extensively investigated to achieve precise control in recent years.

Parameter control for the evolving printing process is shifting from empirical formulas to data-driven, intelligent strategies. Closed-loop control and in-situ monitoring have been proven to effectively enhance printing stability and consistency. ML enables new avenues for parameter decoupling, which is critical for achieving path compensation in challenging scenarios, including work on insulating substrates and curved surfaces. In material engineering, the development of functional inks has pivoted to a multi-pronged strategy. This encompasses the quantitative regulation of rheological and physical properties, the development of eco-friendly and biocompatible solvents, and the formulation of low-dimensional nanomaterials into printable inks. All are directed toward fulfilling the demand for higher performance and multifunctionality. On the hardware front, nozzle design has evolved beyond conventional single-needle configurations toward advanced systems, such as multi-nozzle arrays. These innovations aim at the precise control of spatial electric fields, which is essential to suppress random jet oscillation and boost printing throughput. However, despite the proliferation of research and the availability of numerous reviews covering various aspects of EHD printing, a systematic synthesis of these specific control strategies and their underlying physical correlations remains absent.

This review synthesizes recent advances in performance control strategies for EHD printing. “EHD printing mechanism” examines the mechanisms governing cone-jet formation and provides a critical evaluation of various theoretical models. “High-resolution controllable printing and prediction” focuses on methodologies for achieving high-resolution controllable printing, with a critical evaluation of the roles played by process parameter regulation, functional ink formulation, and nozzle design in enhancing printing stability, precision, and efficiency. “EHD printing applications” highlights the latest applications of EHD printing in diverse fields, including electronic devices, biomedicine, optics, and energy harvesting devices. Finally, we analyze the prevailing technical challenges and outline promising avenues for future development.

## EHD printing mechanism

### Printing mode and jet evolution

**Fig. 1:**
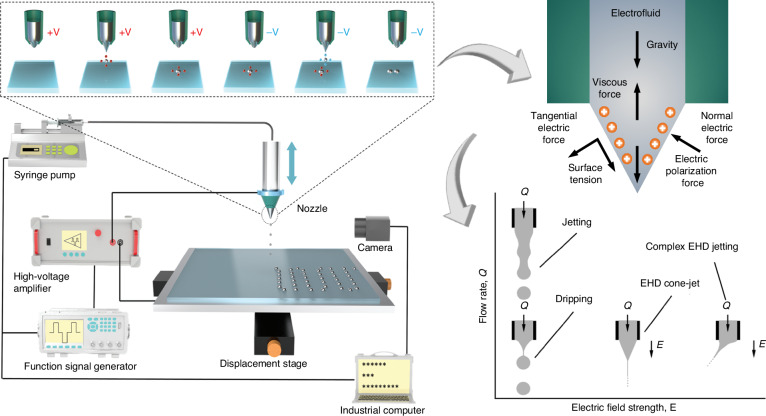
**Schematic diagram of the EHD printing principle**^27,73^. Copyright 2007 Cambridge University Press and 2025 John Wiley & Sons

The printing system is composed of a voltage system, displacement stage, nozzle, syringe pump, camera, and computer, as shown in Fig. 1. The voltage system consists of a signal generator and a voltage amplifier to generate a high-voltage electric field between the nozzle and substrate, while the syringe pump precisely controls the ink flow rate supplied to the nozzle tip. The displacement stage enables precise movement of the substrate along the horizontal and the nozzle along the vertical, thereby achieving patterned deposition of droplets. This is combined with real-time observation of jet morphology and droplet deposition through the camera.

The electrofluid is subjected to the combined effects of gravity, surface tension, viscous force, and electric field force during the printing process^25^. Under the influence of the high-voltage electric field, mobile ions in the functional ink gradually accumulate on the surface of the pendant droplet to form the meniscus. Shear stress generated by charge accumulation stretches the droplet into a conical shape, known as the Taylor cone^26^. When the meniscus potential exceeds the critical value, the electric field force and gravity overcome surface tension and viscous resistance, causing the Taylor cone to emit much smaller than the nozzle’s inner diameter toward the substrate. At this point, the computer controls the displacement stage according to the preset pattern to precisely deposit droplets at designated positions.

**Fig. 2:**
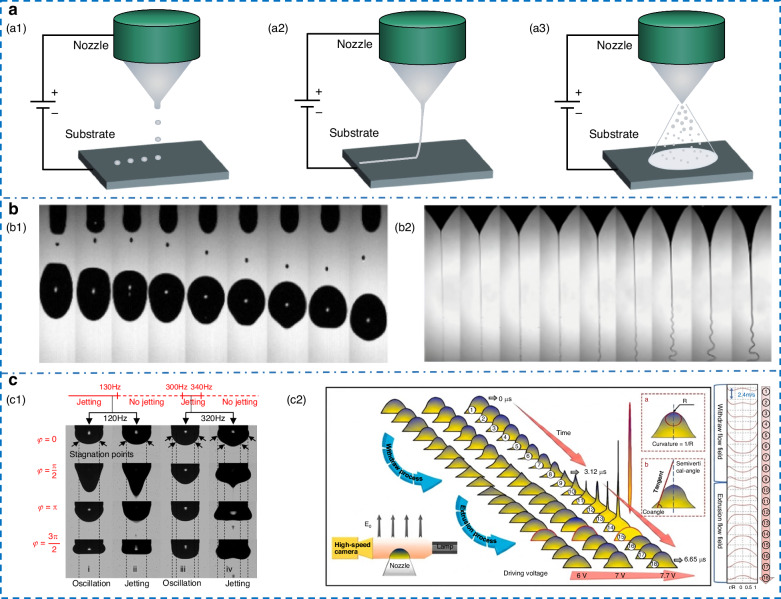
**Research on the EHD printing mechanism.** **a** Three printing mode. **a1** DOD. **a2** Electrospinning. **a3** Electrospray. **b** Printing instability. **b1** Satellite droplets^42^. **b2** Whipping^37^. Copyright 2015 American Institute of Physics and 2022 Elsevier. **c** Residual oscillation phenomenon. **c1** Relationship between frequency and jetting under residual oscillation^54^. Copyright 2021 American Institute of Physics. **c2** Interface creep behavior of MVEHD-jet^57^. Copyright 2025 Elsevier

EHD jets display a range of jetting behaviors, which are influenced by the ink’s physical properties and the system’s parameters. Collins’ team^27^ developed a systematic classification of four distinct modes (Fig. 1). At low electric field strengths and small flow rates, the electrostatic force and surface tension establish a quasi-static balance. In this case, the droplets are released in a dripping mode that is primarily governed by gravity. As the electric field intensity increases, a stable Taylor cone forms at the nozzle tip to produce regular and periodic pulsations. When the applied voltage reaches the critical value, the jet enters an axially symmetric stable cone-jet. Beyond this threshold, the equilibrium among multiple forces is disrupted to generate complex behaviors, such as oblique jet or split jet. The cone-jet configuration has received the most attention because of its high precision and controllability. Cone-jet can be further classified into three forms based on droplet morphology: drop-on-demand (DOD), electrospinning, and electrospray^28^ (Fig. 2a). The details of the three modes are presented in Table 2. DOD produces structured deposits via controlled droplets or jets, typically employing low-pulse voltage with a short nozzle-substrate distance^29^. When the system operates at a larger working distance, polymer solutions are ejected to form highly porous fibers with diameters ranging from tens of nanometers to several micrometers, the process known as electrospinning, widely applied in biomedical and composite materials^30^. When charged droplets attain the Rayleigh limit, they fission and self-disperse via Coulombic repulsion, leading to a uniform, high-velocity charged spray. Electrospray typically employs low-viscosity or high-conductivity small-molecule inks, generating particles at the sub-micron scale, mainly used in thin-film, nanoparticle, and biomolecular^31^.

**Table 2:** Characteristics of EHD printing

Mode	Form	Size	Characteristic	Mechanism
DOD	Droplet	<50 μm	Deposition enables the fabrication of complex patterns	Transient coupling between the pulsed electric field and the mechanical equilibrium of the meniscus
Electrospinning	Fiber	50 nm–10 μm	High surface areas and aspect ratios enable directional alignment and 3D stacking	Intense jet stretching and thinning occur, with polymer chain entanglements resisting fragmentation
Electrospray	Aerosol	<1 μm	Uniform nanoparticles enable the formation of large-area dense thin films	Coulombic explosion triggers droplet breakup once the charge reaches the Rayleigh limit

The insufficient understanding of mechanisms limited the development of this technology. The coupling effects of nozzle, substrate, ink properties, and applied voltage collectively determine jet ejection. Characterizing the complex rheological behavior of two-phase flow has become the pivotal challenge in EHD research. In 2013, Lee et al.^32^ proposed six dimensionless parameters to systematically describe printing behavior, mapped phase diagrams of different jetting modes as functions of flow rate and voltage, providing an important theoretical framework. However, they overlooked the influence of voltage duration, limiting its accuracy in describing transient processes. Therefore, later studies developed more complex multi-physics transient models.

While electric fields can reduce droplet size, their quantitative role in governing droplet diameter and generation frequency during periodic dripping, along with how droplet charge relates to the Rayleigh limit, calls for a deeper understanding. Wang et al.^33^ established a dimensionless scaling law (based on the electric Bond number) that relates droplet diameter to applied voltage and fluid properties, though it applies only to Newtonian fluids. Working within the leaky dielectric model and using the electric Bond number as the key criterion, Guan et al.^34^ proposed a charge-driven pulsation mechanism and corresponding frequency scaling laws. This reveals a fundamental “charge accumulation-ejection-retraction” cycle at low electric Bond numbers, while at high frequencies, the pulsation rate is controlled by the charge relaxation time.

The leaky dielectric model neglects possible transient bulk charges, which may form during high-frequency pulsation if the charge relaxation time is comparable to the pulsation period. To explore new electrospray modes, they introduced the electric Bond number and a dimensionless flow rate. By normalizing the Taylor cone morphology, jet breakup length, and oscillation frequency, the rotational and pulsating atomization regimes have been defined and identified nozzle height as the decisive factor for atomization coverage^35^. Absence of a unified mathematical framework for liquid surface instability patterns motivated Li et al.^36^ to propose a structural function method based on Bessel function superposition. Their approach provides a unified description for diverse patterns, from concentric rings to rose windows. Nevertheless, 2D models are inherently limited to capturing the 3D topology of dot-like patterns, and the underlying symmetry-breaking mechanisms remainfor further study.

### Theoretical model of printing stability

Improving jet stability remains a persistent research priority in EHD printing. Jet instabilities primarily arise from the interplay between surface tension and electric field forces, manifested mainly in two modes: satellite droplets and jet whipping. In addition, the residual oscillations in the meniscus cannot be ignored either. These mechanisms jointly govern jet breakup behavior and printing quality^37,38^. This section will respectively summarize the latest progress in these instability theories, as shown in Table 3.

**Table 3:** EHD printing stability theoretical model

Purpose	Scope	Limitation	Ref.
Effects of nozzle geometry on atomization mode	Atomization modes of low-viscosity Newtonian fluids	Uncertainty remains in models for predicting the atomization area	^35^
Instability patterns lack a unified mathematical description	Pattern formation under unipolar charge injection	Kinematic descriptions fail to predict the dynamic evolution of patterns	^36^
Mechanisms governing the transitions between electrospray modes.	Prediction of the stable operating window for the cone-jet mode	2D axisymmetric models fail to simulate multi-jet modes	^38^
Droplet formation under pulsed voltage excitation	DOD mode	Neglect of abrupt viscosity variations induced by solvent evaporation	^44^
Prediction of 3D nonlinear breakup in finite-conductivity droplets	Non-axisymmetric deformation of fluids	High computational cost	^45^
Onset mechanisms of whipping instability	Critical operating regimes for the whipping instability mode	Neglect of bulk charge effects	^49^
Unified critical criteria for whipping instability	Prediction of the stable operating window for electrospinning	Simplified aerodynamic drag models	^51^
Printing instability and non-uniformity induced by residual oscillation	DOD modes demanding stringent consistency	Requirement to readjust the suppression waveform amplitude in response to ejection voltage variations	^55^
Printing frequency constrained by the intrinsic capillary frequency	DOD and pinch-off mode	Susceptibility to droplet coalescence and merging phenomena in the long-jet regime	^56^

#### Satellite droplet

Although the physical framework is relatively well established, suppressing satellite droplets remains a critical challenge for high-resolution printing (Fig. 2b1). Early studies attempted to eliminate instability through ink-property modulation, but due to limited mechanistic understanding, such strategies often lacked universality^39^. Later research shifted toward in-depth quantitative analysis of breakup mechanisms. The mechanism of satellite droplet formation via nonlinear Plateau-Rayleigh instability in conductor-pure jets was first elucidated through boundary element method simulations and high-speed camera imaging, yet real-world fluids are often markedly more complex^40–42^.

In 2023, Gong et al.^43^ uncovered the Plateau-Rayleigh instability of satellite droplets under pulsed voltage, achieving satellite-free jetting through bias-voltage and viscosity modulation. Xue et al.^44^ showed that in pulsed modes, the duty cycle drives satellite droplet generation: excessive pulse width triggers jet tail breakup. A higher voltage was found to yield a smaller droplet radius, attributing this effect to the emergence of multi-jet or finer single-jet states at elevated voltages. In response to predictive inaccuracies in EHD simulations arising from 2D limitations and neglected charge convection, Nabila et al.^45^ developed a fully 3D Level Set framework that accounts for finite electric Reynolds numbers. This framework allowed the construction of a droplet breakup mode phase diagram, yielding a new theoretical basis for satellite droplet elimination.

#### Whipping instability

Whipping instability represents another major challenge that limits both resolution and structural consistency. It is commonly observed in electrospinning, where jets exhibit helical instability under high-voltage fields (Fig. 2b2). Key strategies for suppression include determining the whipping onset position and extending the stable jet length. Early studies showed that reducing electrode spacing enhanced control over fiber formation. Inspired by this, the near-field electrospinning process was formally proposed, reducing the working distance from the centimeter to the millimeter scale^46,47^. This effectively suppressed whipping instability while lowering operating voltage, though the reduced working distance slightly decreased printing resolution.

As the primary theoretical model for whipping instability, the discrete bead-chain model works by discretizing the continuous jet into point masses. This establishes a dimensionless link between fluid properties and the instability onset location, enabling efficient prediction of the stable jet length^48^. This model, however, neglects internal flow dynamics and thus cannot explain the instability’s origin. With recent advances in computing, the focus has now shifted decisively toward building full 3D computational frameworks.

To address unknowns in whipping instability onset and 3D evolution, Guan et al.^49,50^ studied the varicose-whipping transition, identifying conductivity and flow rate as the key controls, not voltage. The mechanism shows that instability is preceded by uneven surface charge, which asymmetrically stretches the jet from the higher-density side. While their stability map matches experiments well, the use of ethanol limits applicability, as electrospinning typically employs non-Newtonian polymers whose complex rheology poses a continued challenge for prediction. Furthermore, utilizing a one-dimensional flow hypothesis, Xue et al.^51^ established the stress ratio as a unified criterion for the whipping instability threshold. Their work quantified the dual influence of viscosity on jet stability, shedding new light on the development of theoretical models.

#### Residual oscillation

Residual energy in the meniscus induces periodic oscillations that decay gradually when the voltage is turned off, and printing stops, and the printing quality will be severely degraded if the next cycle starts before full decay^52^. Eliminating meniscus residual oscillations is essential for high-frequency EHD printing. The most straightforward approach is to increase the inter-pulse interval to allow natural decay, but it conflicts with high-frequency requirements. Jets achieve higher frequencies under overdamped conditions due to viscous forces effectively dissipating residual energy^53^. Building on this, Li et al.^54,55^ analyzed the quantitative relationship between oscillation and voltage (Fig. 2c1), establishing a forced-damping model to guide pulse voltage design. Reverse-polarity pulse was applied in accordance with the oscillation evolution pattern to cancel residual energy, increasing the frequency limit by over 2.6 times. However, these methods cannot fully overcome the limitation imposed by the meniscus’s inherent capillary frequency.

The research focus has shifted from suppressing to utilizing oscillations. In 2025, Wang et al.^56^ coupled the Poisson-Nernst-Planck equation with the phase-field method, constructing a transition function to resolve numerical instability from charge relaxation effects in LDM. The high-frequency printing mechanism of continuous pinch-off jets was revealed, and a parameter-independent metric for printing frequency was developed. By examining the effects of conductivity, viscosity, flow rate, and voltage on frequency, the optimal process window predicts the highest frequency up to three times the capillary frequency. In the same year, Li et al.^57^ proposed a new printing method based on meniscus vibration, systematically elucidating the mechanisms of forced surface vibration, cone sharpening, and jet formation (Fig. 2c2). By driving meniscus resonance via piezoelectric ceramics coupled, the jetting cycle was compressed to the nanosecond scale, thus achieving megahertz-level printing and improving 2–4 orders of magnitude over traditional EHD printing.

## High-resolution controllable printing and prediction

### Parameter regulation

The core aim in regulating is to achieve stable, high-frequency, and high-resolution printing. Such performance is also the key requirement for large-scale automated production^58^. Due to the complex coupling of multiple physical phenomena in two-phase flow, researchers put forward various strategies for process control. These strategies are drawn from multiple perspectives and include closed-loop control, numerical simulation, machine learning, etc., as outlined in Table 4.

**Table 4:** Technological parameter control strategies

Method	Resolution	Printing mode	Substrate	Ink	Viscosity (cPs)	Ref.
Closed-loop control	15 μm	Cone-jet	Si	PEO	/	^64^
Numerical simulation	1 μm	Electrospinning	Nylon	Polystyrene, PU	/	^80^
ML	100 ppi	Cone-jet	Pixel pit	Epoxy	22	^70,71,73,82^
	100 nm	Cone-jet	/	PEO, ethanol PEDOT, PSS	/	
	15 μm	DOD	/	HIL, HTL, EML	/	
	30 μm	Pulsating jet	Glass	Nano-silver	9.4	
Waveform modulation	5.3 μm	Cone-jet	SiO_2_	AgNPs UV	6-10 12	^82^
Plasma induction	450 nm	Cone-jet	/	Triethylene glycol	49	^85,86^
	3 μm	Electrospinning	Glass, PDMS	PEO, perovskite		
Curved surface	144 µm	Cone-jet	plastic film on a conductive base	Ag, carbon	10,000	^87,91,92^
	<10 µm	Electrospinning, electrospray	Micro-cylindrical	AgNPs, PtNPs, CNTs PEDOT: PSS		
	10 μm	Cone-jet	Ceramic, quartz, plastic	Pt, Ag, CNT	100-1000	

#### Closed-loop control

Conventional EHD printing predominantly adopts open-loop control, which faces the challenge of robustness and stability due to environmental disturbances and ink variation. Closed-loop control addresses this problem by relying on efficient algorithms and accurate state perception as its foundational cornerstones.

In 2010, Mishra et al.^59^ pioneered the integration of ILC into printing processes, adjusting pulsed DC voltage to compensate for changes in working conditions. Programmable control of droplet size and spacing has been achieved at 1 kHz. In 2023, Faejam et al.^60^ introduced a signal-shaping framework based on the P-type ILC algorithm, which further enhances the precise regulation capability over frequency and volume during continuous printing.

Zahra et al.^61^ developed a multi-input multi-output spatial iterative learning control framework for improved geometric accuracy and surface finish in multi-material 3D structures. Separately, Angelo et al.^62^ focused on a process-driven strategy, optimizing the input voltage waveform to reduce nozzle clogging. Mohammadi et al.^63^ developed a rigorous, physics-based state-space model for EHD dynamics. Their model employs nested feedback loops to progressively constrain system states, ensuring the Taylor cone remains in the stable cone-jet regime. The study is limited, however, as it does not analyze the coupling between saturation and system dynamics or discuss anti-windup compensation strategies.

**Fig. 3:**
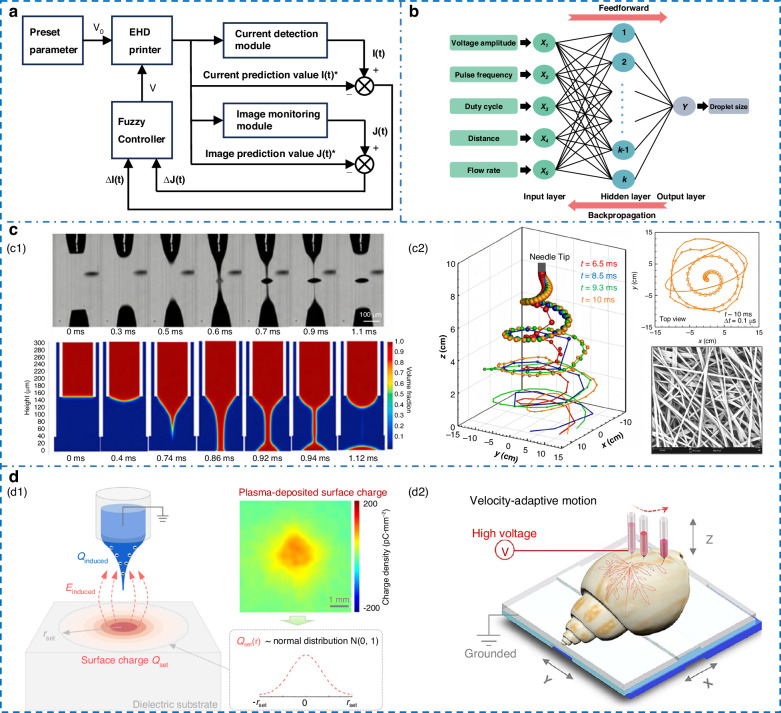
**Printing parameter control methods.** **a** EHD printing fuzzy control closed-loop system^64^. Copyright 2025 Springer Nature. **b** Topological structure schematic of parameter optimization ANN^73^. Copyright 2025 John Wiley & Sons. **c** Application of numerical method. **c1** Comparison between simulation and experiment of the droplet generation process^74^. Copyright 2021 Elsevier. **c2** 3D whipping simulation^78^. Copyright 2024 AIP Publishing LLC. d Printing on special substrate. **d1** Surface charge-induced printing on insulating substrate^86^. Copyright 2024 AIP Publishing LLC. **d2** Freeform curved surfaces^94^. Copyright 2025 American Chemical Society

Closed-loop control based solely on vision or current feedback faces inherent limitations: vision is plagued by millisecond latency, while current signals are noisy and lack detail. The key to efficient real-time control therefore, is a system that can harness both the rapid response of current and the morphological richness of vision. Addressing this challenge, Liu et al.^64^ developed a dual-loop fuzzy control system that separately acquires electrical and image signals (Fig. 3a). Their system uses a fuzzy PID algorithm with current and image-feature deviations as inputs to dynamically regulate the voltage, allowing for prolonged stable printing. ML constitutes another effective approach for integrating such multimodal signals, detailed later in “Machine learning”.

#### Machine learning

ML shows significant potential in EHD printing. This technology can extract effective features from unstructured datasets generated in the printing process and establish mappings without explicit mathematical equations for droplet volume prediction and parameter assistance. Meanwhile, rapid advances in the chip industry provide robust hardware support^65,66^.

The optimization and prediction of process parameters is the predominant application of ML in EHD printing. By building complex mappings between multi-dimensional parameters and printing outcomes, these models allow for bidirectional prediction. Furthermore, they can be paired with global optimizers like genetic algorithms to rapidly identify optimal parameter sets, drastically cutting development costs and improving optimization efficiency. For microgravity environments where experimental data are scarce, Wolf et al.^67^ proposed a heterogeneous transfer model leveraging abundant terrestrial data and sparse flight data to predict printed line dimensions accurately, though its interpolation-based design confines predictions to established parameter bounds. In a complementary approach, Cheng et al.^68^ integrated backpropagation neural networks with genetic algorithms to accurately predict Taylor cone geometry from process inputs. Xu et al.^65^ employed artificial neural networks to map process parameters to droplet diameters, using combined numerical and experimental data. They then coupled this model with a genetic algorithm, enabling the inverse design of process parameters for targeted droplet sizes.

Real-time, closed-loop control of printing systems via ML remains a pivotal and high-value challenge. ML excels at extracting features from low-resolution images, enabling micron-scale monitoring with low-cost cameras. Moreover, lightweight models can theoretically detect jet anomalies within milliseconds. This capability, combined with the immediate feedback they provide, paves a viable path toward large-scale industrialization^69^. To overcome limitations of predictions based solely on process parameters, Zhao et al.^70^ integrated Taylor cone image features with process parameters by a multi-source data model, achieving 92% online prediction accuracy in pixel-pit substrate experiments. Similarly, Jiang et al.^71^ implemented in-situ monitoring with a low-cost optical system to analyze Taylor cone behavior in real time, thereby facilitating indirect tracking of micro-droplet dynamics. A multi-objective stochastic programming model based on uncertainty sets was established by Wang et al.^72^, achieving an 80% improvement in uniformity under a rolling update strategy. However, the learning process is prone to becoming trapped in local optima or experiencing convergence failure. In 2025, Liu et al.^73^ recognized the superior global optimization capability of the EHO algorithm and proposed an intelligent optimization framework for AC pulse modulated parameters using EHO-ANN prediction models, achieving droplet size control on insulating substrates, as shown in Fig. 3b.

#### Numerical simulation

Numerical simulation is an effective tool to reduce experimental costs and capture field distributions at specific times, as a critical tool for investigating jet behaviors. Advances in computing power and numerical methods have matured simulation into a key tool for probing EHD micro-physics and optimizing experiments. Techniques such as the Level Set and Finite Element Methods empower researchers to compute electric field distributions for complex electrodes and trace the dynamic transition from dripping to cone-jet and multi-jet modes. This allows for the efficient, low-cost verification of multi-parameter interactions, thereby elucidating the intrinsic link between process parameters and jet behavior and paving the way for effective control strategies.

As shown in Fig. 3c, the simulation captures four stages: formation, jet ejection, breakup, and droplet deposition^74^. However, despite their sophistication, current simulations remain limited when handling scenarios that more closely approximate actual manufacturing. The 2D axisymmetric assumption simplifies the geometry, drastically reducing mesh counts and enabling high-precision, cost-effective simulation of steady Taylor cone shapes and micro-flow fields^75^. To mitigate the pervasive issue of numerical charge leakage in VOF methods, Julian et al.^76^ developed an extended OpenFOAM solver within this framework. By incorporating a compressive term into the charge transport equation to confine charges at the interface, significantly improved the accuracy of deposited volume predictions. While Kofi et al.^77^ developed a hybrid weighted-averaging scheme to address poor interface prediction in traditional methods, Guo et al.^38^ built a 2D axisymmetric model to uncover the physics behind electrospray mode transitions and their parametric dependences. However, the enforced rotational symmetry in these models precludes the analysis of complex 3D phenomena, such as whipping instabilities and lateral Coulombic interference in multi-nozzle setups.

While computationally expensive, 3D models are essential to fully capture the 3D topology of space charge and the jet’s nonlinear dynamics, overcoming the physical distortions of 2D simplifications (Fig. 3c2)^78^. Cândido et al.^79^ pioneered a 3D framework linking electric potential to whipping instability, with simulated Taylor cones matching experiments well. Guan et al. extended this approach with a full 3D model to analyze how key parameters govern the varicose-whipping transition, validating scaling laws against experimental data^50^. Despite this validation, the substantial computational demand of such models remains a major constraint. Hybrid Lagrangian-Eulerian methods reduce computational demands by, for example, coupling CFD and DEM, as demonstrated by Rahman et al.^80^. This coupled framework achieves precise prediction of fiber diameter and deposition spread while avoiding the mesh explosion problem inherent in VOF-based simulation of microscopic jets.

#### Insulating and curved substrate

Traditional EHD printing typically uses flat conductive substrates, which limit material compatibility, structural adaptability, and functional integration. With the diversification of micro/nanoscale functional devices, high-resolution printing on insulating curved substrates has become crucial.

Insulating substrates will be polarization because the residual charges from droplets cannot dissipate quickly. neutralization of residual charge via oppositely polarized droplets has been demonstrated as an effective approach to eliminate electrostatic repulsion^81,82^. To enable stable high-frequency printing on flexible insulators, Chen et al.^83^ superimposed an AC voltage on pulsed waveforms, neutralizing space charge and accelerating the Taylor cone cycle. In contrast, to tackle the lack of methods for 3D insulating structures, Bai et al.^84^ developed a bipolar EHD jetting strategy. This achieved void-free filling of high-aspect-ratio through-glass vias, showcasing the strategy’s superior performance on strongly insulating substrates. Plasma induction is an emerging technology with great promise for EHD printing on insulating substrates (Fig. 3d1). By directing a stream of high-energy ions toward the surface, it neutralizes residual charges, effectively suppressing electrostatic accumulation on the dielectric interface. Subsequent jets are self-aligned onto existing microstructures, enabling high-precision patterning on glass or flexible polymers without requiring a conductive grounding layer. However, extended plasma exposure can degrade both substrate and ink, which has so far confined experimental validation to relatively stable polymers like PEO^85,86^.

Non-uniform spatial electric-field distribution caused by surface morphology is the core challenge in curved-substrate printing, where mere parameter adjustment yields unsatisfactory outcomes. Integrating EHD direct writing with etching offers a potential solution. To improve interfacial conductivity and mechanical stability, Jung et al.^87^ formulated both the ink and electrodes with a shared silver base. This design, combined with the use of perpendicular electric field lines on conductive curved surfaces, enabled accurate vertical deposition and stacking. Nevertheless, the dependence on noble metals incurs substantial fabrication costs. Employing multi-axis motion platforms for active compensation of errors induced by surface topography has emerged as a robust alternative^88–90^. In 2025, Lai et al.^91^ designed a 4-DOF printing system coupling object rotation and translation to address mapping errors and stress concentration on microcylinders. By coordinating the nozzle with a rotating stage, Wu et al.^92^ addressed issues of interlayer bonding and shape fidelity, proving the feasibility of vertically stacking devices on fibers. However, the high precision required for rotation and the use of only uniform micro-cylinders.

Combining EHD printing with etching techniques opens a new route to fabrication. Ge et al.^93^ developed a 6-DOF multi-robot system that performs surface pretreatment, conformal printing, and in-situ sintering. Using lithography as a guide instead of direct ink deposition, it reaches 5 μm resolution and 35 μm repeatability across large, complex 3D surfaces. Algorithmic correction proves viable for negating the adverse impacts of surface conductivity and geometry. Yue et al.^94^ scanned 3D point clouds to reconstruct the surface and introduced a velocity‑modulation algorithm based on Gaussian curvature (Fig. 3d2). By dynamically adjusting the nozzle speed and compensating for conductivity in real time, they printed circuits with highly uniform line‑width on freeform surfaces, withstanding extreme curvature gradients from 10 to 2000 m⁻¹.

### Function ink control

Printing ink is composed of functional materials, polymers, solvents, and additives. Its physical and chemical characteristics, such as viscosity, surface tension, and conductivity, exert a critical influence on jet formation and the final print quality. Proper formulation is key to suppressing the coffee-ring effect while achieving high-resolution and stable printing^95–97^.

#### Ink physical properties

The physical properties of a functional ink underpin the stable formation of the Taylor cone in EHD printing. Conductivity dictates the surface charge density and tangential electric force, driving jet initiation, whereas surface tension acts as the opposing force to maintain equilibrium. Their interplay defines the stable operating voltage and flow rate range. Furthermore, viscosity and dielectric constant synergistically influence the charge relaxation time.

**Fig. 4:**
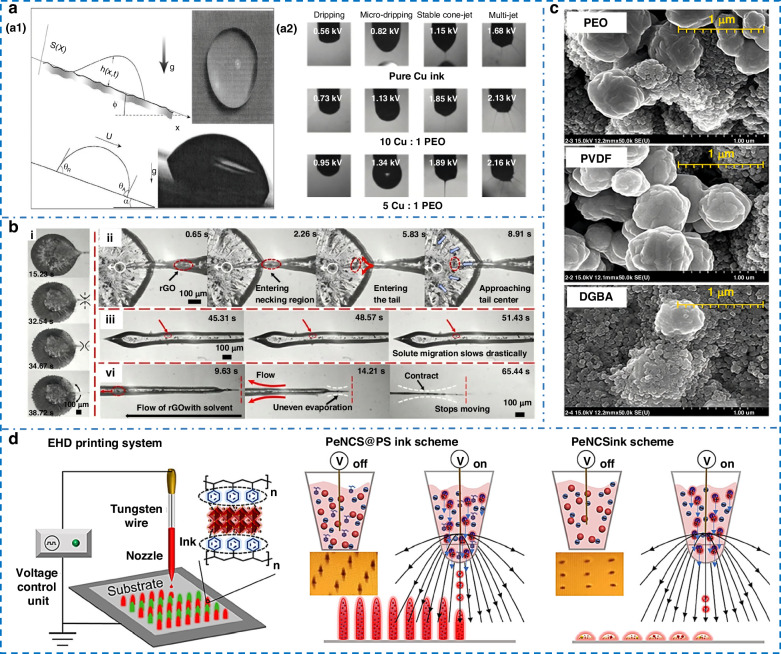
**The influence of ink properties.** **a** Jet and droplets’ behaviors under different viscosities. **a1** low viscosity droplets on inclined substrate^90^. Copyright 2020 John Wiley & Sons. **a2** Critical voltage for printing mode conversion^101^. Copyright 2020 Elsevier. **b** Analysis of sol migration and structural evolution^96^. Copyright 2025 Elsevier. **c** SEM images of different ink components^105^. Copyright 2022 Elsevier. **d** Printing with ink containing nanoparticles^117^. Copyright 2024 Wiley Online Library

Current EHD printing systems commonly use inks with viscosities ranging from 1 to 12,000 cPs, much higher than conventional inkjet printing. Low viscosity means droplets cannot resist jet-induced spreading and may slide or roll on uneven substrates (Fig. 4a1). Droplet volume increases with viscosity, while excessive viscosity may compromise stability. Similar to inkjet printing, nozzle clogging poses a challenge in EHD, particularly when working with high-viscosity nanoparticle inks^98–100^. Li et al.^101^ optimized the formulation of copper/PEO composite electrode lines to adjust the base ink’s viscosity, achieving stable cone-jet configurations with well-defined edges. The use of low-viscosity, toxic toluene solvents in 2D nanosheet inks promotes re-aggregation and sedimentation, undermining printing stability. Rijo et al.^102^ combated this by formulating inks with eco-friendly solvents and ethyl cellulose thickeners. Raising the matrix viscosity to 4000 cPs effectively suppressed nanosheet aggregation and ensured robust deposition.

EHD printing fundamentally involves electric-field forces overcoming surface tension and other resisting forces. Surface tension directly affects critical voltage, stability, and wettability^103,104^. High surface tension increases the energy required to initiate jetting, while its reduction tends to promote the formation of satellite droplets. Ahn et al.^105^ developed temperature-sensitive inks based on polymer solutions (Fig. 4c). The coffee-ring effect is successfully suppressed by incorporating DGBA additives. Inks with high surface tension suffer from dewetting on hydrophobic substrates, preventing continuous circuit formation. Ren et al.^106^ resolved this by blending surfactants into aqueous silver nanowire inks, lowering their static/dynamic surface tension to match the PDMS substrate. This enabled direct printing of serpentine conductive patterns without prior surface treatment. Bai et al.^107^ modulated the PWM duty cycle to control the effective electric field energy density at the Taylor cone, providing stronger electrostatic traction for high-surface-tension inks while suppressing satellites in low-surface-tension inks. This strategy significantly broadened the printable surface-tension window without requiring ink reformulation.

Conductivity directly affects the relaxation time and tangential electric stresses. Low conductivity necessitates higher voltages to overcome slow charge relaxation but risks instability, whereas excessively high conductivity may lead to multi-jet. Printing voltage is also correlated with conductivity. High-conductivity solutions transition to a micro-dripping mode at lower voltages, enabling high-frequency pulsed operation, while excessively high conductivity may induce jet bifurcation. Ink formulations typically control carrier ion concentration using dilute acids or salt solutions, while the minimum flow rate required for stable conical jet formation is independent of conductivity^108–110^.

#### Rheological regulation

Under high-voltage fields, micro-jets experience extreme stretching and shear, making the ink’s dynamic rheology a critical factor. The viscoelasticity of polymer inks, particularly strain-hardening, counteracts Rayleigh-Plateau instability. While long polymer chains stabilize the jet, the resulting high internal friction significantly increases the required voltage. Strong viscoelasticity also triggers tailing, which limits both printing speed and pattern fidelity^111^.

By co-optimizing solvent composition and process parameters, Hassan et al.^112^ achieved a stable cone-jet mode even in viscoelastic-dominated regimes. For polymer inks, however, viscoelasticity causes a tailing ligament to form during droplet breakup, which impairs the uniformity of printed dot arrays. Gong et al.^113^ established how pulsed voltage parameters correlate with ink relaxation time, showing that tailoring the pulse falling edge adjusts the elastic retraction force to counteract viscoelastic tailing. Nevertheless, the finite relaxation time of viscoelastic fluids continues to restrict the maximum printing frequency, keeping it substantially below that achievable with Newtonian fluids.

Adding nanoparticles endows the ink with strong shear-thinning behavior. The high shear rate in the nozzle aligns particles, triggering a viscosity drop that eases jetting. Upon deposition, viscosity swiftly recovers, locking in the shape of the printed micro/nanostructures. While beneficial for performance, high solid loading promotes particle agglomeration and nozzle clogging. This trade-off underscores that understanding the link between inter-particle forces and non-Newtonian rheological thresholds is key to effective ink design. Li et al. solved the dispersion problem of carbon black ink by encapsulating the particles with surfactants to form a homogeneous suspension in ethanol/deionized water^114,115^. Meanwhile, Kirscht et al.^116^ developed a maskless synthesis method regulated by pH that decouples the effects of particle size and organic concentration, overcoming the performance trade-off between conductivity and flexibility in printed thin films.

#### Solvent engineering

The evaporation kinetics of the solvent dictate the liquid-to-solid phase transition path, ultimately defining the final morphology. This process is accelerated by the inherently large surface area of jets, which can induce rapid drying that leads to nozzle clogging or coffee-ring effects.

EHD 3D printing faces a dilemma in ink formulation. On one hand, adding polymer binders for viscosity introduces organic residues that impair conductivity after sintering. On the other hand, pure nanoparticle inks are prone to coffee-ring effects and cannot form high-aspect-ratio, self-supporting features. Developing binary solvent systems has therefore emerged as an effective solution path. Binary solvent systems use a high-boiling-point component to prevent clogging and a low-boiling-point one to drive rapid phase change at the cone tip. Lin et al.^117^ developed a non-polar system that protects perovskite quantum dots from degradation while maintaining print quality. However, two major challenges persist: process instability due to solvent drift, and the fundamental trade-off between conductivity and solubility, as shown in Fig. 4d.

To address stability issues in solvent-based EHD printing, Alberto et al.^118^ systematically studied how molecular weight and evaporation rate affect the process. They found that while higher molecular weight provides greater resistance to jet stretching, it also makes the process more vulnerable to solvent loss, thus creating a critical trade-off. To reduce volatility and nozzle clogging in PLGA inks, Seo et al.^119^ added glycerol as an evaporation suppressant. Through formulation optimization, they achieved a blend that retained stable physical properties for six hours. While effective, the biological impact of residual glycerol was not assessed. An additional challenge in embedded EHD printing is that low-viscosity inks often suffer from solute migration during drying, leading to non-uniform structures. Tan et al.^96^ applied the Young-Laplace equation to analyze pressure differences from uneven line width, establishing a mechanism and map for solute migration and deposition. This enabled the printing of uniform rGO/ethanol lines on liquid substrates. Given that ethanol’s high volatility drives the migration, the map’s validity for low-volatility solvents needs verification.

### Nozzle design

Downsizing nozzles improves resolution in single-nozzle EHD printing, but achieving both high resolution and high throughput remains a major challenge due to drastically increased flow resistance, corona discharge, and clogging with nanoparticle inks. Two main strategies are being explored: using parallel nozzle arrays to boost throughput, and applying auxiliary electric fields to focus the jet^120^. The statistical results of printing performance for different structures are summarized in Table 5. These approaches enhance precision, efficiency, or material adaptability through energy control, reactive deposition, and gravity compensation, collectively shaping a multi-process-integrated EHD printing landscape.

**Table 5:** Comparison of different EHD printing nozzles and system designs

Structure	Resolution	Printing mode	Substrate	Ink	Viscosity (cPs)	Ref.
Multi-nozzle arrays	200 μm	Pulsating jet	SCS	/	/	^125^
	45 μm	Cone-jet	ITO glass	TEG	50	^126^
	10 μm	DOD	Glass, flexible polymer	Ethanol, ethylene glycol	1, 40	^127^
Dual ring-electrode	6 μm	Pulsating jet	/	Ethanol, octanol	1.2–8.9	^135^
Coaxial printing with double tip focus	100 nm	Cone-jet	Silicon dioxide	PZT, silicon oil	160–58560	^138^
Laser assistance	180 nm	Cone-jet	/	ZnO	/	^118^
	604 nm	DOD	PET film, glass	Gold nanoparticle	/	^154^
Supersonic vibration	1 μm	Cone-jet	Glass	PEO	10000	^146^
Redox printing	250 nm	EHD-RP	Metal, insulator	Acetonitrile	/	^144^
	50 nm	EHD-RP	Au, Si	Acetonitrile	/	^149^

#### Multi-nozzle array

Scaling up nozzle arrays represents a direct strategy to enhance throughput and facilitate industrialization. Early work realized synchronized multi-jet control and parallel material printing via nozzle arrays. However, escalating nozzle density exacerbates electric field crosstalk, significantly undermining both printing precision and system stability^121–123^.

**Fig. 5:**
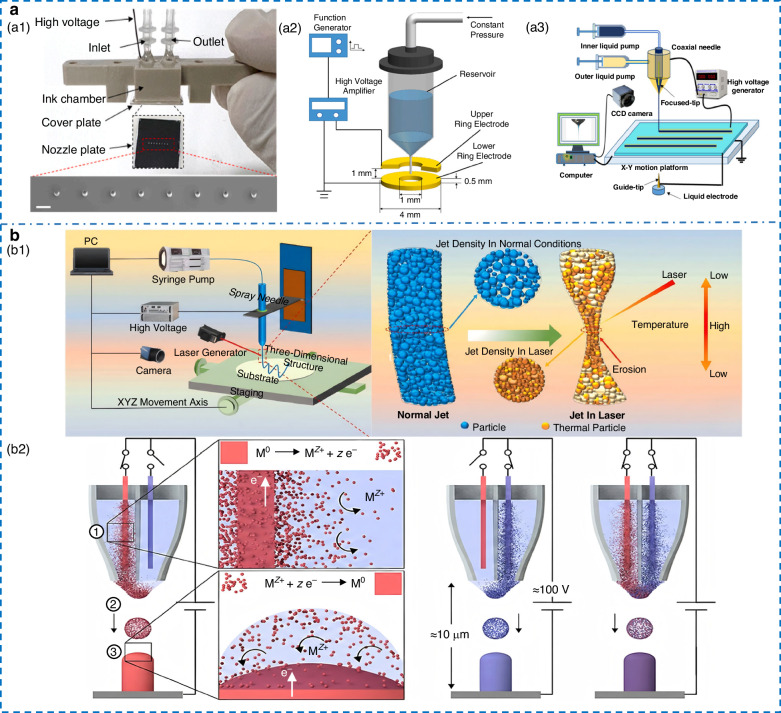
**Schematic diagrams of different configuration printing devices.** **a** Mainstream structural improvement. **a1** Multi-nozzle array^126^. Copyright 2023 Elsevier. **a2** Dual-ring electrode design^135^. Copyright 2023 Elsevier. **a3** Coaxial printing system with dual-focusing tips^138^. Copyright 2024 Springer Nature. **b** Combine other printing principles. **b1** Laser-assisted printing^118^. Copyright 2024 Elsevier. **b2** redox printing^144^. Copyright 2019 Springer Nature

Electric field crosstalk originates from the superposition of electrostatic fields, causing local jet distortion. In closely spaced arrays, Coulombic repulsion between jets deviates them from their intended positions, and electrostatic shielding from neighboring nozzles weakens the central field, further raising the required onset voltage for central nozzles^124^. In 2022, Peng et al.^125^ developed simulation models to quantify how dummy-nozzle density influences crosstalk suppression efficiency. In 2023, Yang et al.^126^ proposed design principles favoring low-conductivity materials and large spacing (Fig. 5a1), and established a working phase diagram for crosstalk levels, but only applicable to conductive substrates. By proposing geometric design rules to suppress crosstalk and using polymer nozzles to prevent breakdown and flooding, Duan et al.^127^ integrated and independently controlled 256 nozzles, reaching 23 kHz. They project that scaling nozzles to 15 μm could push frequencies over 100 kHz, paving the way for industrial-scale production.

Although configuring virtual nozzles addresses electric field crosstalk, it does so at the cost of active nozzle density. To maintain both print quality and throughput, some researchers have pursued active trajectory control by applying differentiated, dynamic voltages to individual nozzles. Using shielding plates is a common yet limited active control method; it struggles with small-angle deflections and lacks strong control over the jet’s flight path^128^. In response, Wen et al.^129^ proposed a new approach that integrates electric field confinement with focusing. A confinement mesh electrode placed below the nozzle produces a toroidal electric field that constrains the jet against lateral deviation. Simultaneously, a needle-shaped electrode aligned coaxially with the nozzle beneath the substrate actively directs the jet to the intended point. This method provides strong crosstalk suppression, reducing deviations from edge effects to 2%, though current electrode alignment challenges limit the nozzle spacing to 1 mm. Chen et al.^130^ treated electric field crosstalk not as a nuisance but as a functional tool. They balanced the Coulombic repulsion between adjacent menisci by tuning the voltages on glass nozzles, enabling parallel printing of two materials. This dynamic equilibrium is delicate, suggesting that future applications will likely require closed-loop feedback to overcome inherent open-loop instability.

#### External auxiliary electrode

Jet focusing via external electric fields can effectively enhance printing precision; two typical designs are the ring electrode and focusing tip (Fig. 5a2). Introducing the electrode ring between the nozzle and substrate to generate radially converging fields strengthens the field gradient at the nozzle tip to overcome height-dependent limitations in 3D printing^131,132^.

Noting that current research pays limited attention to controlling droplet morphology in flight, Xu et al.^133^ integrated a low-voltage ring electrode below the nozzle. The electric field forces from this electrode decelerate the primary droplet, inducing the trailing filament to retract and coalesce. This reduces the breakup length and ensures satellite-free deposition. In conventional systems, printing multi-layer or 3D curved structures causes height variations, which disrupt the electric field and jet stability. To solve this, Seo et al.^119^ built a ring electrode system with a rotary stage, applying an incrementally rising pulse voltage to suppress satellite droplets. This enabled precise patterning of drug arrays on cylindrical surfaces.

Although the single-ring electrode has demonstrated printing capabilities comparable to conventional systems, its relatively simple structure results in a narrow operating parameter window and limited control over the electric field. The adoption of a dual-ring electrode configuration to restructure the built-in focusing electric field has been proven to be a reliable optimization strategy. Two coaxial ring electrodes can significantly enhance jet stability. Independently adjustable inner and outer ring voltages enable finer electric field control, facilitating higher-frequency pulse operation^134,135^. Bai et al. investigated the dual-ring electrode mechanism by analyzing how printing height, voltage, and electrode diameter affect jetting. They pinpointed the onset voltage-jet velocity relationship and showed that a smaller bottom ring sharpens field focusing. A notable limitation is the Newtonian fluid assumption, which overlooks how viscoelasticity influences breakup^136^.

The focusing tips embedded in the nozzle significantly enhances the local field^137^. In 2024, Zou et al.^23^ established a tip-assisted focused printing system that produced 2.3 μm droplet arrays and microwires, nearly quintupling resolution. Same year, Shi et al.^138^ proposed dual-tip-assisted printing (Fig. 5a3), which not only increased the tip-field strength but also reduced reflux, making it possible to deposit 100 nm conductive lines directly on SiO₂ substrate. Furthermore, they combined two auxiliary electrodes: an internal tungsten tip to locally boost the field for ejecting viscous ink, and an external ring electrode to stabilize the field against substrate variations. This integrated approach improved printing uniformity and successfully produced high-performance, biomimetic flexible pressure sensors^139^.

#### Coaxial printing

Coaxial EHD printing utilizes a nested nozzle configuration to encapsulate a core fluid within a sheath fluid. Leveraging shear forces from the sheath significantly lowers the jetting threshold for the functional core material. This approach fundamentally overcomes the limitations of conventional single-nozzle systems, and its unique capability to fabricate core-shell architectures has driven widespread adoption across many frontier domains.

Coaxial nozzles reduce clogging via ink circulation, but controlling the ink bridge between capillaries often yields larger, less stable features than single nozzles. To stabilize it, Li et al.^140^ built a pressure-driven coaxial system with closed-loop feedback. It analyzes real-time images of the meniscus and dynamically adjusts the vacuum to regulate the compound Taylor cone’s shape, ensuring long-term stability. Their validation focused on DOD mode; whether it can stabilize continuous jetting remains untested. Onyekuru et al.^141^ aimed at evaluating the necessity of coaxial over uniaxial processing by comparing electrospun and electrosprayed model proteins from both systems. The coaxial electrospraying yielded samples with the lowest enzymatic activity. This finding demonstrates that in many cases, the simpler uniaxial method can effectively replace the more complex coaxial approach.

Correlating real-time microcurrent signals with droplet behavior decoded the current pulses during ejection and retraction. This analysis identified voltage and core flow rate as stability keys: within the optimal window, raising both improved resolutions, producing satellite‑free core‑shell droplet arrays. The logical next step is to evolve this post‑analysis model for Newtonian fluids into a real‑time, closed‑loop system for non‑Newtonian inks^142,143^.

#### Other printing system structures

Beyond mainstream optimizations of electrical and geometric parameters, EHD printing systems exhibit exceptional structural flexibility tailored to specific application scenarios. Various auxiliary energy fields and structural innovations have been integrated to expand capabilities (Fig. 5b), e.g., redox printing^144^, laser-assisted printing^145^, ultrasonic vibration printing^146^, electrostatic-assisted dispensing^147^, and counter-gravity printing^114,148^, significantly expanding the process boundaries of the technology.

To overcome the limited control over local chemistry in microscale additive manufacturing, Reiser et al.^144^ introduced electrohydrodynamic redox printing (EHD‑RP). It works by dissolving a sacrificial anode into metal ions, jetting them to the substrate, where they are reduced to deposit pure metal. This unique approach eliminates both ink formulation and post‑processing, and enables rapid material switching and alloying from a single nozzle. Maxence et al.^149^ precisely modulated printed feature dimensions by controlling solvent evaporation and droplet Coulombic explosion, determining 50 nm to be the resolution limit in stable stacking mode. Porenta et al.^150^ fabricated nanoporous materials to investigate the microstructural basis of mechanical properties. Their results showed that the inherent layer‑by‑layer deposition of EHD‑RP forms a more ordered hierarchical network, giving the samples significantly greater hardness than those produced by magnetron sputtering.

EHD printing, which uses electric fields rather than gravity to eject fluid, is ideal for space manufacturing. Huang et al.^151^ explored this capability in parabolic-flight microgravity. They found that gas bubbles, unable to escape without buoyancy, created a unique nanoporous structure in printed ZnO. Notably, both the operating voltage and power consumption were slashed compared to Earth-based fabrication. Jiang et al.^152^ showed sensors made in microgravity perform robustly, but at the cost of higher voltages to compensate for the lack of gravity and maintain resolution. The underlying mechanisms are not fully understood, and current studies rely on brief weightlessness simulations (e.g., parabolic flights), making further investigation in authentic microgravity essential.

Auxiliary energy fields, such as lasers or heat, offer a solution for printing high‑viscosity inks by dynamically tuning local rheology through multi‑physics coupling. Li et al. developed various laser‑assisted EHD printing strategies that utilize the localized surface plasmon resonance of gold nanoparticles to absorb light and generate in‑flight heating. This allows for non‑destructive, low‑temperature manufacturing on low‑melting‑point substrates and single‑step integration of micro/nanostructures. However, precisely focusing the laser onto the micron‑scale Taylor cone poses a formidable challenge for scale‑up, as the process is extremely sensitive to minute environmental fluctuations^118,153,154^. To create in‑situ porosity within fibers, Jia et al.^155^ added in‑process heating, using high temperatures to boil solvent and pyrolyze polymer. Jiang^146^ integrated an ultrasonic transducer; its vibrations lower wall friction and apparent viscosity, enabling stable jetting of viscous ink without air pressure. Since high‑frequency vibration causes viscous heating and the transducer itself generates heat, a more comprehensive understanding would require coupled thermal‑field analysis in future work.

## EHD printing applications

EHD printing offers high resolution, broad material compatibility, and excellent conformability to flexible or non-planar substrates, enabling it to transcend the limits of conventional photolithography and inkjet processes and advance from laboratory research to integrated device development. This section, therefore, reviews key application breakthroughs: electronics, biomedicine, optics and energy harvesting. Detailed parameters of printing products are provided in Table 6.

**Table 6:** Performance of devices manufactured by EHD printing

Time	Product	Resolution	Ink	Function	Ref.
Electronic device	E-nose	20 μm	Pd-SnO_2_, Pd-WO_3_	The power of 17 mW can reach 300 °C, and the accuracy rate of 8 kinds of gas is 99.86%, 30-day stability error <10%	^159^
	H_2_S gas sensor	1.1 μm	SnO_2_/Pd@TiO_2_	Monitoring limits 6 ppb, 5ppm H_2_S response value 17.9, response attenuates 10.6% in 9 months	^160^
	OFET	90 nm	IDT-BT, F8-BT	Mobility ratio 1.1 cm² V^−1^ s^−1^, switch ratio 1.93 × 10⁵	^135^
	QLED	2540 ppi	Teflon, QD	External quantum efficiency 20.29%, luminance 35,816 cd m^−2^	^168^
	Micro-OLEDs	3600 ppi	HIL, HTL, EML	Printing layer high uniformity 96.3%	^169^
Biomedicine	Sinusoidal scaffold	10 μm	PCL	Enhancing cardiomyocyte contractility, Young’s modulus 0.35 MPa	^172^
	NGC	48.6 ± 4.5 μm	PCL, PEO, MWCNTs	Myelinated axon density 7206 ± 456 nm^−2^, muscle wet weight ratio 65.2 ± 3.2%	^174^
	Tri-layered scaffold	25.7 ± 5.1 μm	PCL, PEO, SDF-1	Maximum failure load 150.97 ± 8.25 N, cell recruitment density ~1998 cells mm^−2^	^175^
	SPN	50 nm	HAS, salt methanol	Targeted drug delivery in nanomedicine	^178^
Energy harvesting device	Electrode	12.8 μm	ZIF-67, PCL	Specific capacitance 230 mF/g	^64^
	MSC	80 μm	MXene	Volumetric capacitance 2013 F cm^−3^, and energy density 100.7 mWh cm^−3^	^181^
	TENG	/	PAM, CNF, MXene	Detection range 1% to 550%, open circuit voltage 67.5 V	^184^
Optical device	Microlense	/	Nematic liquid crystal	Polarization-dependent focusing, Focal distance shift ~0.4 mm)	^186^
	Resonator	260 nm	Glycerol, ethanol, UV	Temperature sensitivity 0.142 nm/°C, magnetic field sensitivity 8.63 pm/mT	^187^

### Electronic device

Leveraging its core advantages of high resolution and stability at low cost, EHD printing has evolved into one of the fundamental processes for micro/nano electronic manufacturing. Flexible electronics, displays, and sensors can all be rapidly prototyped through direct writing. While EHD printing can produce conductive patterns and traces at the nanoscale, this capability does not directly translate to the resolution achievable in final electronic products^156^.

Sensors fabricated via EHD printing exhibit exceptional comprehensive performance and operational stability. These sensor arrays demonstrate broad substrate adaptability, spanning from rigid to flexible platforms, while enabling single or multi-parameter integration for detecting temperature, humidity, gas, and other variables. Notably, sensors manufactured with specialized alloy materials can even exhibit self-recovery capabilities under specific conditions^157,158^. In 2023, Li et al.^159^ combined laser curing to integrate dual sensing units on a single chip, demonstrating excellent conformal-surface characteristics and narrow-cavity monitoring. Based on dual-arm suspended-bridge microthermal plates, the system achieved 300 °C operating temperature at 17 mW power consumption with 30-day stability errors below 10%. To overcome limitations of traditional single-layer structures, Li et al.^160^ in 2024 designed a three-layer H₂S sensor using in-situ preparation: SnO₂ as the electron-transport layer, Pd/TiO₂ as the coating layer, and Sn-Ti as the transition layer. This configuration improved sensitivity by 6.8 times compared with single-layer SnO₂, achieving a detection limit of 6 ppb with only 10.6% response decay over nine months. A novel solution for monitoring trace H₂S in enclosed environments (e.g., spacecraft) and industrial applications was enhanced by this innovation. Shi et al.^139^ developed a biomimetic flexible piezoresistive sensor with a sensitivity of 25.18 kPa⁻¹. It showed remarkable durability, enduring 1000 cycles at 5 kPa with no signs of fatigue, making it suitable for monitoring human motion and health. Dávila et al.^161^ doped waveguide structures with fluorescent materials, presenting a novel strategy for the low-cost industrial manufacturing of flexible optical sensors.

**Fig. 6:**
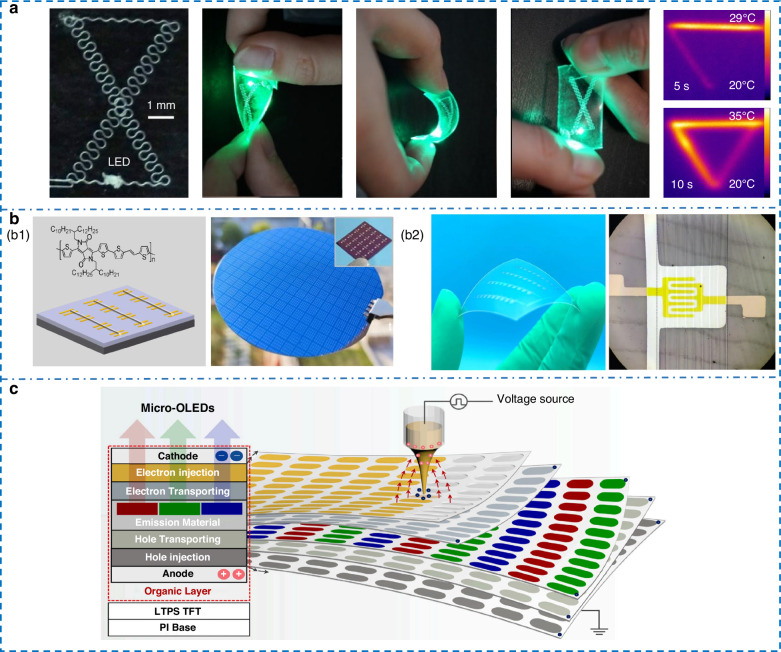
**Applications in electronic devices.** **a** Stability and thermal imaging of vertically interconnected two-layer circuits^163^. Copyright 2021 John Wiley & Sons. **b** Manufacturing of transistors. **b1** OFET structure based on sub-micron lines and performance curves^164^. Copyright 2022 Springer Nature. **b2** Flexible OFET^135^. Copyright 2023 Springer Nature. **c** Trilayer structures of Micro-QLED manufacturing^169^. Copyright 2025 Elsevier

EHD printing offers distinct benefits in the manufacturing of integrated and functional electronic devices. It enables the co-fabrication of active driving units (e.g., TFT backplanes) and light-emitting display components (QLED or OLED) within displays; however, their performance objectives tend to diverge. While transistors, typically comprising integrated multilayered heterostructures, prioritize interlayer alignment precision and interfacial control, display technologies aim for uniform deposition across large-area patterns. EHD printed TFTs demonstrate outstanding switch ratios and charge carrier mobility. Transistors formed by line arrays with diameters less than 100 nm exhibit an average mobility approaching 2 cm^2^ V⁻¹ s⁻¹. Directly printed flexible vertical interconnects enable the realization of bendable and wearable electronic devices. Incorporating protective architectures, such as those utilizing epoxy resins, further enhances reliability under bending and tensile stress, illustrated by the stable electrical response shown in Fig. 6 across 40% tensile strain^162–165^.

EHD printing emerges as one of the most promising approaches for future display manufacturing. As early as 2015, Rogers’ team^166^ successfully produced quantum dot patterns with a line width of 400 nm, demonstrating performance comparable to devices fabricated via spin-coating or vacuum deposition. Its low-temperature characteristics also offer an effective pathway to address thermal management challenges inherent in conventional methods. In 2023, Wang et al.^167^ fabricated dual-color QLEDs with 500 ppi resolution, reaching a maximum current efficiency of 16.5 cd A^−1^ for the top emission devices. In 2024, Yang et al.^168^ implemented a Teflon mesh patterned atop a spin-coated quantum dot layer, leveraging its insulating properties to prevent direct contact between the hole transport layer and electron transport layer. This approach effectively addressed pixel leakage issues while maintaining quantum dot luminescent efficiency, achieving a remarkable brightness of 35,816 cd m^−2^. For Micro-OLED multilayered structures, which often suffer from printing defects due to underlying charge deposition and electric field crosstalk during fabrication, Zhao et al.^169^ introduced a deep reinforcement learning-based closed-loop control system in 2025. Their method enabled precise printing of HIL/HTL/EML trilayer structures onto pixel pit substrates (Fig. 6c), attaining a layer height uniformity of 96.3% and a record resolution exceeding 3600 ppi, which meets the needs for commercial mass production of VR/AR display panels.

### Biomedicine

Conventional biomanufacturing techniques are constrained by limitations such as mechanical contact damage, poor material compatibility, and insufficient structural resolution, making it difficult to meet the personalized demands of complex biomedical devices. In contrast, EHD printing enables precise manipulation of bioink microflows, facilitating cross-scale fabrication from DNA printing to biomimetic scaffolds. This approach offers novel solutions for cell culture, tissue engineering, and smart drug delivery systems.

**Fig. 7:**
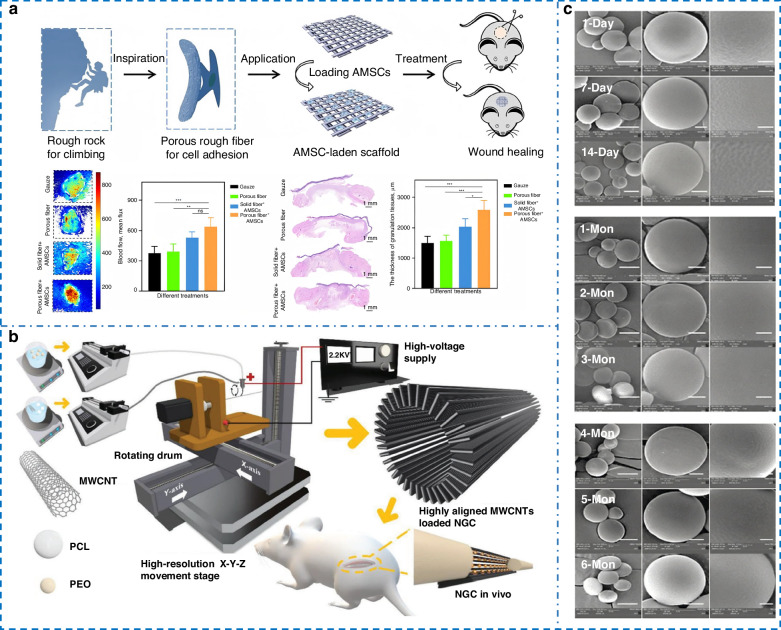
**Applications in biomedicine.** **a** Mesenchymal stem cell therapy: Scaffold structure inspired by rock climbing and comparison of wound healing effects^170^. Copyright 2022 Springer Nature. **b** Schematic for printing nerve guidance conduits with oriented coaxial fibers^175^. Copyright 2024 John Wiley & Sons. **c** Morphological changes of spherical shell particles during six months of in vitro release^176^. Copyright 2022 Elsevier

Huang et al.^170^ pioneered an EHD cryoprinting technique, where glacial acetic acid dissolution of PCL fibers increased surface roughness. The comparative effect of the treatment is shown in Fig. 7a. Mesenchymal stem cells are easier to adhere to the sites and facilitate fibroblast migration and vascular endothelial cell tubule formation. In 2024, Wang et al.^171^ immobilized basic fibroblast growth factor on PCL scaffolds using PDA chemistry. Triggering tenogenic differentiation requires both topographic and chemical cues, and appropriate immunomodulation was conducive to its growth. Conventional tissue scaffolds made of straight fibers limit cardiomyocyte contraction and hinder tissue maturation. To overcome this, Lei et al.^172^ harnessed the mechanical buckling of an impinging jet to create self‑assembled sinusoidal patterns. The resulting wavy, biomimetic scaffold closely resembles native collagen architecture, effectively promoting cell alignment and maturation.

Most EHD‑printed scaffolds have only single‑scale fiber architectures, limiting their ability to guide cell behavior. To create a more biomimetic environment, Zhang et al.^173^ fabricated a hierarchical micro‑scaffold that mimics the natural ECM. It showed excellent stability in water and supported significantly higher cell density and proliferation than pure micro‑scale scaffolds, demonstrating the clear advantage of multiscale design for enhancing cellular response. Bai et al.^174^ created a tri‑layer scaffold that mimics the graded tendon‑to‑bone interface, featuring micro‑scale core‑shell fibers with graded porosity. SDF‑1 from the shell recruit cells quickly, while core‑loaded differentiation factors are released slowly over 4 weeks. This spatiotemporal control boosted target gene expression by up to sevenfold. A major drawback of artificial nerve guidance conduits is their poor support for long‑distance nerve regeneration. To enhance performance, Sun et al.^175^ employed coaxial printing to make core‑shell fibers with integrated MWCNTs. The conduits demonstrated excellent biocompatibility and mechanical properties. Notably, a 1% MWCNT content yielded regeneration comparable to autografts. Increasing the concentration to 1.5%, however, significantly reduced cell viability, defining a practical limit for improving electrical.

Programmable drug structures are also a significant application of EHD printing in the biological field. Tang et al.^176^ used coaxial printing with PLA as the core to develop sustainable releasing spherical shell microparticles. The release duration could be tuned by modifying shell thickness and composition, extending up to six months (Fig. 7b). Although biodegradable polymers (e.g., PLGA) show promise for drug delivery, their direct-writing applications have been limited due to ink volatility causing instability. In 2024, Seo et al.^119^ introduced 10% glycerol into PLGA/DMSO to suppress solvent evaporation. Through pulse voltage modulation to eliminate satellite droplets and precise control of pattern size and density, they achieved controllable drug release. Liu et al.^177^ loaded Yoda1 onto polydopamine (PDA)-modified scaffolds. Leveraging PDA’s chemosorption ensured stable drug loading and sustained release, providing an effective local delivery strategy for bone defects. Separately, to push the size limits of protein nanoparticles, Iqbal et al.^178^ used Human Serum Albumin with trace NaCl to tune particle size, yielding biocompatible nanocarriers averaging 61.2 nm. However, Taylor‑cone stability degrades sharply at higher salt concentrations, fundamentally limiting further size reduction via this route.

### Optical and energy harvesting device

**Fig. 8:**
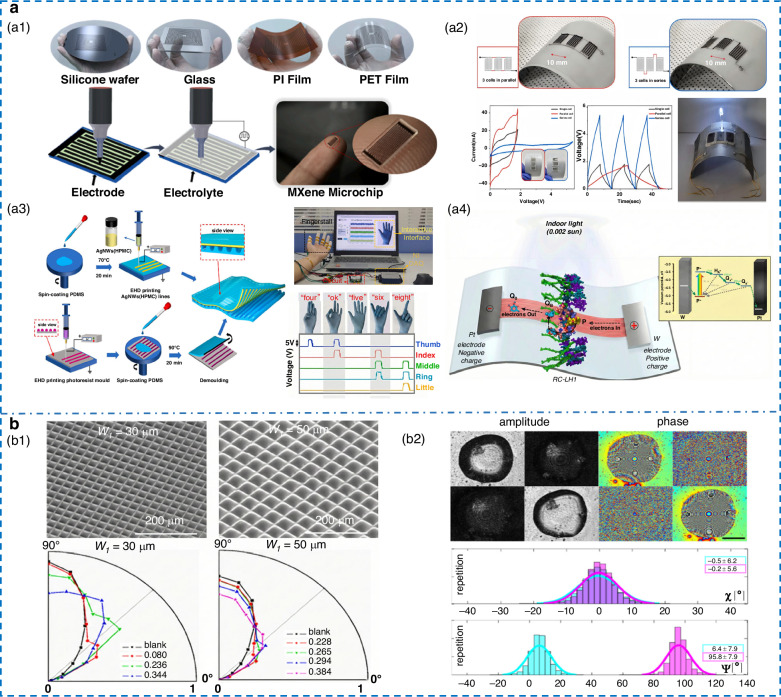
**Applications in energy harvesting and optical devices.** **a** Energy harvesting device. **a1** Printing on different substrates followed by electrolyte deposition, resulting in MXene microchip^181^. Copyright 2026 Elsevier. **a2** MSC on curved substrate^87^. Copyright 2024 Elsevier. **a3** Manufacturing process of the frictional nano generator and gesture recognition test^182^. Copyright 2023 Elsevier. **a4** Schematic diagram of charge separation mechanism in photoelectric capacitors^183^. Copyright 2023 Elsevier. **b** Optical devices. **b1** Self-aligned liquid droplet micro-lens array^185^. Copyright 2020 Elsevier. **b2** Pyro-EHD printed NLC circular lenses^186^. Copyright 2024 American Chemical Society

The micro/nanoscale fabrication capabilities of EHD printing open new opportunities to address bottlenecks in energy harvesting devices and optical devices. Typical energy harvesting devices, such as batteries, supercapacitors, and nanogenerators, have all been developed with complete fabrication methods in the laboratory (Fig. 8a). In 2020, Lee et al.^179^ pioneered the use of EHD printing for MSC, integrating 36 cells on 8 × 8.2 mm² chip surfaces to achieve 65.9 V cm⁻² surface voltage density. Jung et al.^87^ developed chip-level MSCs capable of conforming to complex curved surfaces, precisely creating uniform 3D electrodes across both planar and curved substrates. The structure features a cross-layer homogeneous silver interface, achieving an energy density of 256.6 μWh cm⁻². Liu et al.^180^ engineered flexible supercapacitor electrodes with tunable porosity and ordered grid structures. Increasing the fiber spacing boosts specific capacitance by boosting porosity and enhancing ion access. Recently, Ali et al.^181^ fabricated interdigitated micro‑supercapacitors using a high‑viscosity, organic‑based MXene ink. Despite its complexity, this approach yielded devices with record performance: a volumetric capacitance of 2013 F cm⁻³, a maximum energy density of 100.7 mWh cm⁻³, and remarkable stability over 10,000 cycles.

In 2023, Cheng et al.^182^ proposed a porous-array charge-trap design that TENG with enhanced performance via internal-external friction synergy, achieving a maximum output power of 580 μW and passing gesture-recognition tests with high accuracy. In the same year, Paul et al.^183^ utilized photosynthetic protein complexes to create a solid-state flexible photoelectric micro-capacitor operating under indoor light. Hydrogels’ mechanical fragility and difficulty in forming conductive networks have restricted their use in TENGs. Zeng et al.^184^ broke this barrier with a hybrid strategy combining EHD printing and in‑situ photopolymerization. The resulting devices sensed motions from large bends to subtle pulses, and harvested enough energy to light 80 LEDs, demonstrating viability as wearable power sources. Nevertheless, long‑term hydrogel durability and MXene oxidation in wet environments remain key challenges.

EHD printing has also seen broad adoption in optical devices; high-quality MLA is one of these. Li et al.^185^ achieved self-alignment of droplets via heterogeneous-wetting surfaces, precisely controlling droplet volume to regulate lens morphology while independently adjusting base shape, unit size, and aspect ratio (Fig. 8b1). The result was a self-aligned MLA with 99.3% fill factor and a 49% improvement in OLED light extraction efficiency. These methods typically require expensive equipment and heated nozzles to reduce liquid viscosity during printing. To enable room temperature printing of nematic liquid crystal lenses, Coppola et al.^186^ developed a pyro-EHD printing technique (Fig. 8b2). By creating a temperature gradient on crystals to induce built-in electric fields, they formed Taylor cones from NLC droplets and achieved DOD printing without nozzles or external high-voltage power, effectively overcoming the need for heating to reduce viscosity.

Additionally, photodetectors and optical resonators are also applied. In 2025, Zhang et al.^187^ devised an innovative approach that integrated EHD printing with self-assembly driven by Plateau-Rayleigh instability, effectively accounting for capillary and jetting dynamics to enable the fabrication of functional materials spanning three orders of magnitude in viscosity on diverse substrates. This hybrid strategy resulted in the production of whispering gallery mode optical resonators exhibiting a temperature-sensing sensitivity as high as 0.142 nm °C⁻¹.

## Conclusion and perspective

With its unique field-driven mechanism, ultra-high resolution, and broad material compatibility, EHD printing has emerged as a vital enabler for heterogeneously integrated 3D micro/nanodevices. This review surveys the fundamentals of jetting dynamics and stability. It then delineates control and prediction strategies for high-resolution printing, covering advances in process parameters, ink formulations, and nozzle designs. Finally, it showcases frontier applications in electronics, biomedicine, energy harvesting, and optics, highlighting the technology’s potential to scale from lab to industry. Nevertheless, hurdles persist on the path to full industrialization.

1. Nonlinear control in multi-physics coupled environments remains a critical challenge. Current methods still rely on empirical parameter scans or simplistic data fitting, which lack the closed-loop stability needed for mass production. The solution lies in fusing machine-learning-based predictive models with a dual-loop feedback system that integrates current monitoring and machine vision, enabling real-time prediction and dynamic control of process parameters.
2. The trade-off between ink rheology and device functionality. High-performance inks come at the price of clogging, high voltage, and instability. Low-viscosity alternatives, while easy to jet, suffer from the coffee-ring effect and poor resolution. A persistent research priority is to develop novel ink formulations that decouple rheological constraints from functional requirements while ensuring robust biocompatibility.
3. The physicochemical mismatch at multi‑material interfaces. High‑performance devices must unify macroscopic mechanical strength with microscopic bio/chemical activity. Conventional sequential assembly often leads to poor adhesion and delamination. Research must therefore clarify the mechanisms for in‑situ interfacial fusion and develop hybrid, multi‑modal printing platforms that integrate disparate processes into a single, synergistic workflow.
4. Bridging the lab‑to‑factory gap requires multi‑nozzle arrays for throughput, yet they introduce formidable barriers: electric field crosstalk in dense setups, poor nozzle-to-nozzle uniformity, and insufficient long‑term reliability. These issues stand as the principal roadblocks to integrating EHD printing into industrial lines.

EHD printing holds distinct appeal over conventional techniques due to its multi‑dimensional advantages. Despite existing bottlenecks, the deepening convergence of ML, novel rheological materials, and precision mechatronic systems is poised to redefine the micro/nanofabrication landscape.
